# Proteomic and bioinformatic analyses of proteins in the outer membrane and extracellular compartments and outer membrane vesicles of *Candidatus* Liberibacter species

**DOI:** 10.3389/fmicb.2022.977710

**Published:** 2022-09-26

**Authors:** Yixiao Huang, Fanchao Zhu, Jin Koh, Daniel Stanton, Sixue Chen, Nian Wang

**Affiliations:** ^1^Department of Plant Pathology, Citrus Research and Education Center, Institute of Food and Agricultural Sciences, University of Florida, Lake Alfred, FL, United States; ^2^Proteomics and Mass Spectrometry, Interdisciplinary Center for Biotechnology Research, University of Florida, Gainesville, FL, United States; ^3^Citrus Research and Education Center, Department of Microbiology and Cell Science, Institute of Food and Agricultural Sciences, University of Florida, Lake Alfred, FL, United States

**Keywords:** outer membrane protein, outer membrane vesicle, Liberibacter, citrus, HLB

## Abstract

Citrus Huanglongbing (HLB) is the most devastating citrus disease in the world. *Candidatus* Liberibacter asiaticus (Las) is the prevalent HLB pathogen, which is yet to be cultivated. A recent study demonstrates that Las does not contain pathogenicity factors that are directly responsible for HLB symptoms. Instead, Las triggers systemic and chronic immune responses, representing a pathogen-triggered immune disease. Importantly, overproduction of reactive oxygen species (ROS) causes systemic cell death of phloem tissues, thus causing HLB symptoms. Because Las resides in the phloem tissues, it is expected that phloem cell might recognize outer membrane proteins, outer membrane vesicle (OMV) proteins and extracellular proteins of Las to contribute to the immune responses. Because Las has not been cultivated, we used *Liberibacter crescens* (Lcr) as a surrogate to identify proteins in the OM fraction, OMV proteins and extracellular proteins by liquid chromatography with tandem mass spectrometry (LC–MS/MS). We observed OMVs of Lcr under scanning electron microscope, representing the first experimental evidence that Liberibacter can deliver proteins to the extracellular compartment. In addition, we also further analyzed LC–MS/MS data using bioinformatic tools. Our study provides valuable information regarding the biology of *Ca.* Liberibacter species and identifies many putative proteins that may interact with host proteins in the phloem tissues.

## Introduction

Citrus Huanglongbing (HLB, also known as citrus greening) is the most devastating citrus disease worldwide. It is caused by phloem-colonizing bacteria *Ca. L. asiaticus* (Las), *Ca. L. africanus* (Laf) and *Ca. L. americanus* (Lam, syn. *Ca. L. psyllaurous*; Hansen et al., 2008; Liefting et al., 2009) with Las being the most prevalent. HLB remains the No. 1 challenge for citrus growers despite some progress in HLB management including three-pronged management (Yuan et al., 2021; Alquézar et al., 2022), plant defense inducers (Li et al., 2019), antimicrobials (Akula et al., 2011; Li et al., 2019, 2021), heat treatment Thapa et al., 2021), microbiome manipulation (Riera et al., 2017), growth hormones (Canales et al., 2016; Tang and Vashisth, 2020), and enhanced nutrition programs (Stansly et al., 2014). Owing to the inability to culture the HLB pathogens *in vitro*, the pathogenicity mechanism of HLB remains poorly understood (da Graça et al., 2022; Pandey et al., 2022). Las lacks homologs of known pathogenicity factors that are directly responsible for causing plant disease symptoms (Ma et al., 2022). The pathogenicity factors including *vir* genes from closely related *Agrobacterium* and *Rhizobium* pathogens (Chilton et al., 1982; Kuzmanović et al., 2018) were not identified in Las. Las does not contain type II, III, and IV secretion systems that are commonly involved in bacterial virulence (Duan et al., 2009; Thapa et al., 2020). Other virulence factors including Sec-dependent effectors (SDE) were discovered in Las and proposed to activate disease symptoms (Pitino et al., 2016; Clark et al., 2018; Pang et al., 2020), but none of the SDEs cause HLB symptoms when overexpressed in plants (Ma et al., 2022). Prior work also suggested that Las causes HLB symptoms by phloem blockage resulting from deposition of callose and other phloem proteins (Kim et al., 2009; Achor et al., 2010, 2020; Koh et al., 2012), root decay (Johnson et al., 2014), chloroplast disruption due to excessive starch accumulation in plastids (Gonzalez et al., 2012), metabolic burden (Vasconcelos et al., 2021). However, these observations seem to be the consequence of Las infection, rather than the root cause of HLB disease. It has been recently reported that HLB is a pathogen-triggered immune disease (Ma et al., 2022). Las infection stimulates systemic and chronic immune response in phloem tissues and HLB disease symptoms are caused by systemic cell death of those tissues. This response is instigated primarily through excessive and chronic reactive oxygen species (ROS) production.

Las resides inside the phloem tissues. It is possible that citrus phloem cells might recognize Las *via* typical pathogen-associated molecular patterns (PAMPs) including LPS, peptidoglycan, and flagellin to trigger immune responses. It was reported that Las encodes a flagellin containing a conserved 22 amino acid domain (flg22) that induces immune response (Zou et al., 2012) and *Citrus* species contains functional FLS2 responding to flg22 (Shi et al., 2016). In addition, phloem cells might active the immune responses by recognizing Las proteins on the cell surface, i.e., outer membrane proteins (OMPs), and those which are easily released into the phloem tissues, such as proteins contained in outer membrane vesicles, and putative secreted proteins.

OMPs consist of two kinds of proteins: integral outer membrane proteins and peripheral lipoproteins, and together they comprise approximately 2 to 3% of the bacterial proteins (E-komon et al., 2012; Majewski et al., 2018; Hermansen et al., 2022). Typical integral OMPs have a β-barrel fold and can range in size from 8 to 36 strands with short loops between strands on the periplasmic side and large, extended loops on the extracellular side (Fairman et al., 2011; Doyle and Bernstein, 2019). Larger β-barrels were also found, for example, secretin *st*InvG from *Salmonella enterica* and secretin *ec*GspdD from *Escherichia coli* K12 have 60-stranded β-barrel structure (Worrall et al., 2016; Yan et al., 2017). Most OMPs contain an even number of β-strands arranged in an antiparallel pattern (Fairman et al., 2011). So β-strand structure can be used as an analysis target to identify OMPs. Typical lipoproteins have a protein domain located in the periplasm and a lipid part anchored to the inner leaflet of outer membrane. However, many lipoproteins are surfaced-exposed because they can be assembled in complexes with β-barrel proteins like LptE/LptD or RcsF/OMP in *Escherichia coli* (Konovalova and Silhavy, 2015). Importantly, some OMPs are known to induce immune responses. For instance, bacterial pathogens produce lipoproteins were found to induce apoptosis in THP-1 monocytic cells through human Toll-like receptor–2 (hTLR2; Aliprantis et al., 1999).

OMPs are synthesized by ribosomes in the cytoplasm and transferred into the periplasm by passage through the SecYEG translocon in the inner membrane. OMPs are in unfolded conformations during this stage. Then the OMP precursors interact with periplasmic chaperones to prevent misfolding and can be delivered to the β-barrel assembly machinery (BAM) to be inserted into the outer membrane (Rollauer et al., 2015). OMPs fulfill multiple functions including nutrient uptake, waste export, cell adhesion, and cell communication. Outer membrane proteins such as lipoproteins have been known to activate immune responses in mammalian cells (Hashimoto et al., 2006).

Outer membrane vesicles (OMVs) are spherical membrane-bound structures released from the envelopes of Gram-negative bacteria, ~20–250 nm in diameter. It is postulated that OMVs are produced from the regions of outer membrane where covalent crosslinks between the outer membrane and peptidoglycan decrease (Schwechheimer and Kuehn, 2015). Bacteria can use OMVs to secrete virulence factors into surroundings. OMVs from plant pathogens were reported to induce reactive oxygen species burst and defense-related marker gene expression in *Arabidopsis thaliana* (Bahar et al., 2016). OMVs from *Xanthomonas campestris* pv. vesicatoria, the causal agent of bacterial spot disease in tomato and pepper, contain virulence-associated xylanases and protease (Solé et al., 2015). Bacterial OMVs can also deliver lipopolysaccharide (LPS) into host cell cytosol to activate caspase-11 and immune response (Vanaja et al., 2016).

In this study, we aimed to investigate proteins of the outer membrane fraction, OMV proteins, and putative secreted proteins of *Ca.* Liberibacter. Because only *Liberibacter crescens* (Lcr) is cultured in artificial media and Lcr is of high similarity with other species in the *Liberibacter* genus (Leonard et al., 2012), Lcr BT-1 was used as a surrogate to achieve our goal. Specifically, the 16S rRNA gene of Lcr BT-1 shares 94.7% sequence similarity with the 16S rRNA genes of Lam and Las, 94.0% similarity with Lso, and 93.4% similarity with Laf (Fagen et al., 2014a). The genome size of Lcr is 1.5 MB, which is slightly larger than the ~ 1.2 MB genome of Lam, Las, Lso and Laf (Duan et al., 2009; Lin et al., 2011, 2015; Leonard et al., 2012; Wulff et al., 2014), but the predicted functions encoded by their genomes do not have substantial difference (Fagen et al., 2014b). The shared average nucleotide identity (ANI) between Lcr BT-1 and Las is 77.4% (Fagen et al., 2014a). Las and Lcr encodes 1,183 and 1,380 genes, respectively, with 70% of Las genes having homologs in Lcr (Fagen et al., 2014b). Owing to their similarity, Lcr has been used as a surrogate to investigate the biology of Las (Lai et al., 2016; Jain et al., 2019; Sena-Vélez et al., 2019). In this study, proteins of the outer membrane fraction, OMVs, and extracellular fraction of Lcr BT-1 were extracted and the proteins in these samples were identified by liquid chromatography with tandem mass spectrometry (LC–MS/MS). We used bioinformatic tools to further analyze the LC–MS/MS data. It is anticipated that the information learned from Lcr will shed light on Las and other *Ca.* Liberibacter species.

## Materials and methods

### Bacterial strain and culture conditions

*Liberibacter crescens* BT-1was cultured in Basal Medium 7 (BM7) medium consisting of 2 g alpha-ketoglutarate, 10 g N-(2-Acetamido)-2-aminoethanesulfonic acid (ACES) buffer, 3.75 g KOH, 150 ml of fetal bovine serum (Gibco) and 300 ml of TMN-FH insect medium (Sigma) per litter, adjusted to pH 6.5 (Cruz-Munoz et al., 2019). Bacterial culture was grown at 250 rpm and 28°C. The cultures were routinely tested by PCR (Supplementary Table 1; Jain et al., 2019).

### Isolation of Lcr BT-1 outer membrane fraction

Gram-negative bacteria have two cell membranes with different structures, the cytoplasmic membrane is a phospholipid bilayer while the outer membrane contains phospholipids in the inner leaflet and glycolipids in the outer leaflet (Kleanthous and Armitage, 2015). 0.5% N-Lauroylsarcosine sodium (Sarkosyl) solubilizes cytoplasmic membrane but not the outer membrane of *E. coli* (Filip et al., 1973). Thus, the Sarkosyl solution was used to extract the outer membrane fraction of Lcr. Outer membrane isolation was conducted as described previously (Davise, 1991; Foreman et al., 2010) with modifications. Lcr BT-1 was grown for 7 days for collection at OD_600_ = 0.35. Bacterial cells were collected by centrifugation at 5,000 × *g* for 30 min at 4°C and stored at –70°C in membrane buffer (50 mM sodium phosphate buffer at pH 7.0, 7.5% glycerol, 50 mM NaCl). Then cells were thawed on ice and protease inhibitor cocktail (Roche) was added to the solution. The cells were disrupted using 240 × 5 s of sonication (Misonix Sonicator 3,000 Ultrasonic Cell Disruptor) on ice. Then the sonicated cell slurry was centrifuged at 11,000 × *g* for 10 min at 4°C to remove unbroken cells. The cell envelope was precipitated by ultracentrifugation of 50,000 × *g* for 60 min at 4°C in a Beckman 75 Ti rotor, then resuspended in 0.5% (*w*/*v*) Sarkosyl for 20 min at room temperature to selectively solubilize the cytoplasmic membrane. Another 50,000 × *g* ultracentrifugation to precipitate the remaining outer membrane for 60 min at 4°C. The pellet was washed in 20 mM Tris–HCl (pH 7.2) buffer and centrifuged at 50,000 × *g* for 60 min at 4°C. Finally, the outer membrane was resuspended in 20 mM Tris–HCl (pH 7.2) buffer.

### Isolation of Lcr BT-1 outer membrane vesicle and extracellular proteins

For extraction of outer membrane vesicles and extracellular proteins, Lcr BT-1 was first grown in BM7 medium for 7 days for collection at OD_600_ = 0.35. Lcr cells were collected by centrifugation at 700 × g for 20 min. The pellet was washed using serum-free BM7 medium (BM7 medium without fetal bovine serum) and centrifuged at 700 × g for 20 min for three times. The bacterial cells were then resuspended in serum-free BM7 medium to avoid the interfere on imaging and grown for 2 days.

Outer membrane vesicles (OMVs) were isolated using ExoBacteria^™^ OMV Isolation Kit (System Biosciences) according to the manufacturer’s protocol. The kit used an ion-exchange chromatography system to extract OMVs. Briefly, bacterial culture was centrifuged at 5,000 × *g* for 20 min at 4°C the supernatant was centrifuged again at 5,000 × *g* for 20 min at 4°C to remove cell debris. The supernatant was then filtered through 0.45 μm filter and 0.22 μm filter. At the same time, OMV binding resin was loaded to column the column was equilibrated by flowing through 10 ml binding buffer. Then the bacterial supernatant was added to the column and cap was put on the column. After 30 min incubation on a rotating rack at 4°C, the column was put onto a rack and the bottom and cap of the bottom was removed. After supernatant flowed through the column, the resin was washed with 15 ml Binding buffer three times. Then the resin was incubated with OMV elution buffer for 2 min and the elution buffer was collected in a fresh microcentrifuge tube. OMV samples were then resuspended in 20 mM Tris–HCl (pH 7.2) buffer after acetone precipitation.

Extracellular proteins of Lcr BT-1 were isolated using the trichloroacetic acid (TCA) precipitation method (Koontz, 2014). Lcr cells from serum-free BM7 medium were centrifuged at 5,000 × *g* for 30 min at 4°C and the supernatant was filtered through a 0.22 μm filter. The 10% of culture volume of TCA was added to the filtered supernatant and the solution was kept on ice for 30 min. Then the samples were centrifuged at 10,000 × *g* for 15 min at 4°C. The supernatant was carefully removed, and the pellet was washed with ice-cold acetone. The samples were centrifuged at 10,000 × *g* at 4°C for another 5 min and removed the supernatant. When the pellet dried the samples were resuspended into 20 mM Tris–HCl (pH 7.2) buffer.

### In-solution digestion

Outer membrane fraction, outer membrane vesicle and extracellular protein samples were collected for LC–MS/MS analysis. Each type of samples had three biological replicates. Urea was added to protein samples to a final concentration of 1 M to increase the solubility of proteins. Five microliters (μl) of 200 mM dithiothreitol (DTT) solution were added to solution samples and they were heated up to 95°C for 5 min and incubated at 55°C for additional 45 min. Then the proteins were alkylated by adding 4 μl of 1 M chloroacetamide (CAA) solution and incubated at 25°C for 45 min in darkness. The alkylation was stopped by adding 20 μl DTT solution and incubating the samples at 25°C for 45 min. Trypsin solution was prepared in 50 mM ammonium bicarbonate buffer and added to protein samples to make the final trypsin to protein ratio of 1:50 (*w*/*w*) in solution. The samples were incubated at 37°C for 16 h.

### ZipTip

The resulting peptides from digested protein samples were desalted using micro ZipTip mini-reverse phase (Millipore) with capacity of 2 μg. The ZipTip was first equilibrated with 10 μl of 100% Acetonitrile (ACN), 10 μl of 50% ACN/50% of 0.1% trifluoroacetic acid (TFA) solution, and 10 μl of 0.1% TFA × 3. The suspended peptide sample was pipetted through the ZipTip for ten times, and ZipTip was then again washed with 10 μl of 0.1% TFA for ten times before eluting the sample from the ZipTip with 80% ACN/0.1% TFA solution. The process was repeated for all the samples, and all samples were lyophilized in the SpeedVac.

### Liquid chromatography with tandem mass spectrometry (LC–MS/MS)

Peptides derived from the total proteins were resuspended in 0.1% formic acid. The bottom-up proteomics data acquisition was performed on an EASY-nLC^™^ 1200 ultra-high-performance liquid chromatography system (Thermo Fisher Scientific, Waltham, MA, United States) connected to an Orbitrap Fusion^™^ Tribrid^™^ instrument equipped with a nanoelectrospray source (Thermo Fisher Scientific, Waltham, MA, United States). The peptide samples were loaded into a C18 trapping column (Acclaim^™^ PepMap^™^ 100, 75 μm inner diameter × 2 cm length, 3 μm particle size, and 100 Å pore size) and then eluted using a C18 analytical column (Acclaim^™^ PepMap^™^ 100, 75 μm inner diameter × 15 cm length, 2 μm particle size, and 100 Å pore size). The flow rate was set to 250 nl/min with solvent A (0.1% formic acid in water) and solvent B (0.1% formic acid and 80% ACN) as the mobile phases. The separation was conducted using the following gradient: 2–40% of solvent B over 0–160 min; 40–80% of solvent B over 160–165 min, 80–98% of solvent B over 165–166 min, and kept at 98% of solvent B until 180 min. The column was then thoroughly washed with 98% solvent B and re-equilibrated with 100% solvent A before injection of the next sample.

The full MS1 scan (m/z 350–2,000) was performed on the Orbitrap analyzer with a resolution of 120,000 at m/z 200. The automatic gain control (AGC) target is 2e5 with 50 ms as the maximum injection time. Monoisotopic precursor selection (MIPS) was set to select ions with peptide-like isotopic distributions. Peptides bearing + 2–6 charges were selected with an intensity threshold of 1*e*4. Dynamic exclusion of 15 s was used to prevent resampling the high abundance peptides. Top speed method was used for data dependent acquisition within a cycle of 3 s. The MS/MS was carried out in the linear ion trap, with a quadrupole isolation window of 1.3 Da. Fragmentation of the selected peptides by collision induced dissociation (CID) was done at 35% of normalized collision energy. The MS2 spectra were detected in the linear ion trap with the AGC target as 1*e*4 and the maximum injection time as 35 ms.

### Database searching

All MS/MS samples were analyzed using Mascot (Matrix Science, London, United Kingdom; version 2.7.0.1). Mascot was set up to search the NCBI_Liberibacter_crescens_20220214 database assuming the digestion enzyme trypsin. Mascot was searched with a fragment ion mass tolerance of 1.00 Da and a parent ion tolerance of 10.0 ppm. O + 18 of pyrrolysine and carbamidomethyl of cysteine were specified in Mascot as fixed modifications. Gln- > pyro-Glu of the n-terminus, deamidated of asparagine and glutamine and oxidation of methionine were specified in Mascot as variable modifications.

Scaffold (version Scaffold_4.2.1, Proteome Software Inc., Portland, OR) was used to validate MS/MS based peptide and protein identifications. Peptide identifications were accepted if they could be established at greater than 95.0% probability by the Scaffold Local FDR algorithm. Protein identifications were accepted if they could be established at greater than 95.0% probability and contained at least two identified peptides. Protein probabilities were assigned by the Protein Prophet algorithm (Nesvizhskii et al., 2003). Proteins that contained similar peptides and could not be differentiated based on MS/MS analysis alone were grouped to satisfy the principles of parsimony. Proteins sharing significant peptide evidence were grouped into clusters. The homologs of Lcr proteins in Las were analyzed by BLAST. Las str. psy62 (taxid: 537021) was used as the reference. Subcellular localization of Lcr proteins was predicated using PSORTb (Yu et al., 2010), CELLO (Yu et al., 2004) and SOSUI GtamN (Imai et al., 2008). Predictions by at least two predictors were considered positive for each protein. The subcellular localization of proteins with different prediction results using different predictors were designated as unknown.

### Scanning electron microscopy

Lcr BT-1 cells, OMVs and BM7 medium (control) were observed using scanning electron microscope (SEM). A 20 μl aliquot of the bacteria samples was pipetted on pieces of fractured microscope slides and allowed to dry at room temperature. Samples were fixed in a 4% paraformaldehyde solution buffered with 1x phosphate-buffered saline (PBS) and incubated overnight. The next day samples were dehydrated in an ethanol series (30, 50, 70, 85, 95, and 100%) and then incubated in 100% ethanol overnight at 4°C. The samples were then dried using a Ladd 28,000 critical point dryer (Ladd Research Industries, Williston, VT, United States), mounted on double-sided 12 mm carbon stickers on SEM stubs (Electron Microscopy Sciences, Hatfield, PA, United States), and sputter-coated using a Ladd 30,800 sputter coater (Ladd Research Industries) with a gold/palladium target. Samples were observed using a Hitachi S4000 SEM (Hitachi, Tokyo, Japan) and images were captured with PCI imaging software (Quartz Imaging Corp., Vancouver, BC, United States).

### Bioinformatic analyses

We also conducted bioinformatic analyses for 13 select *Ca.* Liberibacter strains with high-quality complete genomes. These strains include *Lcr* BT-0, *Lcr* BT-1, *Ca. L. solanacearum* str. ZC1, *Ca. L. asiaticus* str. A4, *Ca. L. asiaticus* str. Gxpsy, *Ca. L. asiaticus* str. JRPAMB1, *Ca. L. asiaticus* str. CoFLP, *Ca. L. asiaticus* str. TaiYZ2, *Ca. L. asiaticus* str. psy62, *Ca. L. asiaticus* str. JXGC, *Ca. L. asiaticus* str. Ishi-1, *Ca. L. americanus* str. Sao Pa and *Ca. L. africanus* str. PTSAPSY. The genome sequence of these strains was downloaded from National Center for Biotechnology Information website. OMP prediction of Liberibacter species was conducted using three groups of predictors as describe previously in *Pasteurella multocida* (E-komon et al., 2012). Briefly, PSORTb (Yu et al., 2010), CELLO (Yu et al., 2004) and SOSUI GtamN (Imai et al., 2008) were used as subcellular predictors. Transmembrane beta barrel domains were predicated using TMBETADISC RBF (Ou et al., 2008), BOMP (Berven et al., 2004), and MCMBB (Bagos et al., 2004). Lipoprotein predictors included LIPO (Berven et al., 2006) and LIPOP (Juncker et al., 2003). According to the accuracy, recall/sensitivity, specificity, and Mathews Correlation Coefficient (MCC) analysis on different criteria for consensus prediction of sequences from 526 Gram-negative bacteria proteins with known localization (E-komon et al., 2012), the criteria to predict OMP in this study was decided as follows: For the first two groups of predictors, predictions by at least two were considered positive to be localized in the outer membrane or have a beta-barrel structure. For lipoprotein prediction, it was considered as positive when either one predictor predicts a protein to be a lipoprotein associated with the outer member. After combining results from all these predictors, a protein list of each strain was summarized. Next, we manually verified the annotation of each protein, and CDD was used to analyze conserved domains of target proteins (Lu et al., 2020).

## Results

### Identification of proteins in the *Liberibacter crescens* outer membrane fraction *via* LC–MS/MS

Proteins from Lcr BT-1 outer membrane fraction were identified *via* LC–MS/MS. Proteins identified in all 3 replicates with an average ≥10 spectrum counts/replicate were considered positive, resulting in 55 identified proteins in the outer membrane fraction (Table 1). Among these proteins, 14 were predicted to be OMPs by bioinformatic analyses (Table 2), approximately 30% of the predicated OMPs (14 of 50). In addition, among the rest 35 predicted OMPs, 8 were identified with 5–9 unique spectrum counts, whereas 4 were identified with 1 to 4 unique spectrum counts (Table 2). Proteins identified by LC–MS/MS also included 32 predicated cytoplasmic proteins, 1 extracellular protein, 5 inner membrane proteins, and 2 periplasmic proteins (Table 1). Protein BLAST showed among the 55 proteins identified from Lcr outer membrane fraction, 52 have homolog proteins in Las strains (Table 1).

**Table 1:** Proteins identified from *Liberibacter crescens* BT-1 outer membrane fraction.

Accession Number	Annotation	Subcellular localization prediction	Homolog in Las*
AGA64040.1	Outer membrane protein assembly factor YaeT precursor	Outer membrane	WP_015452389.1
AGA64980.1	RND efflux system, outer membrane lipoprotein CmeC	Outer membrane	WP_015452761.1
AGA64249.1	Heat shock protein 60 family chaperone GroEL	Cytoplasmic	WP_015452683.1
AGA65052.1	Porin	Outer membrane	WP_012778533.1
AGA65276.1	Type II/IV secretion system secretin RcpA/CpaC, Flp pilus assembly	Outer membrane	WP_015452435.1
AGA64650.1	Outer membrane lipoprotein Omp16 precursor	Outer membrane	WP_015452784.1
AGA65195.1	Chaperone protein DnaK	Cytoplasmic	WP_015824904.1
AGA64835.1	DNA-directed RNA polymerase beta’ subunit	Cytoplasmic	WP_012778362.1
AGA65143.1	25 kDa outer-membrane immunogenic protein precursor	Outer membrane	WP_015452561.1
AGA64400.1	Translation elongation factor Tu	Cytoplasmic	WP_012778370.1
AGA64636.1	Kinesin-like protein	Outer membrane	WP_015452861.1
AGA64614.1	hypothetical protein B488_06220	Outer membrane	WP_015452561.1
AGA64343.1	Outer membrane lipoprotein Omp16 precursor	Outer membrane	WP_015452611.1
AGA64846.1	D-3-phosphoglycerate dehydrogenase	Cytoplasmic	ACT56625.1
WP_041770579.1	Protein with unknown function	Cytoplasmic	ACT56857.1
AGA65275.1	Components of type IV pilus	Extracellular	WP_015452436.1
AGA65241.1	HtrA protease/chaperone protein	Outer membrane	WP_015452461.1
AGA64105.1	SSU ribosomal protein S1p	Cytoplasmic	WP_012778675.1
AGA64822.1	Putative ABC Transporter Atp-Binding Protein	Inner membrane	WP_012778359.1
AGA64836.1	DNA-directed RNA polymerase beta subunit	Cytoplasmic	WP_012778363.1
AGA64234.1	Outer membrane protein Imp / Organic solvent tolerance protein precursor	Outer membrane	WP_012778614.1
AGA64028.1	Polyribonucleotide nucleotidyltransferase	Cytoplasmic	WP_012778758.1
AGA64033.1	SSU ribosomal protein S2p (SAe)	Cytoplasmic	WP_015452382.1
AGA64371.1	Glutamine synthetase type I	Cytoplasmic	WP_012778405.1
AGA64013.1	LOW QUALITY PROTEIN: Cyclic beta-1,2-glucan synthase	Inner membrane	absent
AGA64113.1	Aconitate hydratase	Cytoplasmic	WP_012778670.1
AGA64470.1	Omp25	Outer membrane	WP_015452561.1
AGA64823.1	Membrane lipoprotein	Outer membrane	WP_012778361.1
AGA65017.1	Cell division trigger factor	Cytoplasmic	WP_015452726.1
AGA64736.1	Succinyl-CoA ligase (ADP-forming) beta chain	Cytoplasmic	WP_015452880.1
AGA65006.1	Manganese ABC transporter, periplasmic-binding protein SitA	Periplasmic	WP_015452621.1
AGA64399.1	Translation elongation factor G	Cytoplasmic	WP_012778483.1
AGA64275.1	ClpB protein	Cytoplasmic	WP_015452710.1
AGA64110.1	General substrate transporter	Inner membrane	absent
AGA65222.1	NAD-specific glutamate dehydrogenase, large form	Cytoplasmic	WP_015452498.1
AGA64023.1	Translation initiation factor 2	Cytoplasmic	WP_012778755.1
AGA65210.1	NAD-dependent glyceraldehyde-3-phosphate dehydrogenase	Cytoplasmic	WP_015452505.1
AGA64124.1	Carbamoyl-phosphate synthase large chain	Cytoplasmic	WP_012778658.1
AGA65007.1	Manganese ABC transporter, ATP-binding protein SitB	Cytoplasmic	WP_015452620.1
AGA64361.1	NADPH:quinone oxidoreductase 2	Cytoplasmic	absent
AGA65095.1	Dihydrolipoamide acetyltransferase component of pyruvate dehydrogenase complex	Cytoplasmic	WP_171816668.1
AGA64591.1	Competence lipoprotein ComL	Outer membrane	WP_015453002.1
AGA64022.1	Transcription termination protein NusA	Cytoplasmic	WP_050815691.1
AGA64342.1	TolB protein precursor	unknown	WP_015452610.1
AGA64788.1	Zinc ABC transporter, periplasmic-binding protein ZnuA	Periplasmic	WP_015452621.1
AGA64735.1	Succinyl-CoA ligase (ADP-forming) alpha chain	Cytoplasmic	WP_015452881.1
AGA65002.1	3-oxoacyl-(acyl-carrier-protein) synthase, KASII	Cytoplasmic	WP_015452625.1
AGA65274.1	Type II/IV secretion system ATPase TadZ/CpaE, Flp pilus assembly	Cytoplasmic	WP_015452437.1
AGA65260.1	Signal recognition particle, subunit Ffh SRP54	Cytoplasmic	WP_015452450.1
AGA64907.1	ATP-dependent Clp protease ATP-binding subunit ClpX	Cytoplasmic	WP_012778490.1
AGA64987.1	Protein-export membrane protein SecD	Inner membrane	WP_015452756.1
AGA64369.1	ATP-dependent RNA helicase	Cytoplasmic	WP_012778601.1
AGA65313.1	Inner membrane protein translocase component YidC, long form	Inner membrane	WP_012778702.1
AGA65030.1	Inosine-5′-monophosphate dehydrogenase	Cytoplasmic	WP_015452718.1
AGA64820.1	NADP-dependent malic enzyme	Cytoplasmic	WP_012778357.1

* Homolog accession # in Las psy62 was shown.

Subcellular localization prediction was conducted using predictor PSORTb, CELLO, and SOSUI GtamN.

**Table 2:** Bioinformatic prediction of outer membrane proteins in *Liberibacter crescens* BT-1 and the LC–MS/MS results of proteins which were also identified from bacterial outer membrane fraction.

Protein locus	β barrel predictors			Sub-cellular localization predictors			Lipoprotein predictors		LC–MS/MS (spectrum counts in the 3 biological replicates)		
	BOMP	MCMBB	TMBETA DISC-RBF	CELLO	PSORTb	SOSUIGramN	LIPO	LIPOP	1	2	3
AGA63999.1	–	–	–	Y	–	–	Y	–	6	3	6
AGA64002.1	–	Y	Y	Y	–	–	–	–			
AGA64040.1	–	Y	Y	Y	Y	Y	–	–	95	80	96
AGA64234.1	–	Y	Y	Y	Y	–	–	–	23	17	14
AGA64245.1	–	–	Y	–	–	Y	Y	Y			
AGA64332.1	–	Y	Y	Y	–	Y	–	–	1	3	1
AGA64343.1	–	–	Y	Y	Y	–	–	–	27	23	28
AGA64354.1	–	Y	Y	Y	–	–	–	Y	2	2	3
AGA64387.1	Y	Y	–	–	–	–	–	–			
AGA64389.1	Y	Y	Y	Y	Y	–	–	–			
AGA64428.1	–	–	Y	Y	–	Y	–	–			
AGA64431.1	–	–	–	–	–	–	Y	Y			
AGA64432.1	–	–	–	–	–	–	–	Y			
AGA64438.1	–	–	–	–	–	–	Y	–			
AGA64470.1	Y	Y	Y	Y	Y	Y	–	–	13	16	14
AGA64492.1	–	Y	Y	–	–	–	–	–			
AGA64501.1	–	Y	Y	–	–	–	–	–	3	4	3
AGA64506.1	–	Y	Y	Y	–	–	–	–			
AGA64508.1	–	Y	Y	Y	–	Y	–	–			
AGA64510.1	–	Y	Y	–	–	–	–	–			
AGA64549.1	–	–	–	–	–	Y	Y	Y	8	5	6
AGA64564.1	Y	Y	–	–	Y	–	–	–	6	9	3
AGA64573.1	–	–	–	–	Y	Y	Y	Y	3	1	3
AGA64585.1	–	Y	Y	Y	–	–	–	–			
AGA64591.1	–	–	Y	–	Y	–	Y	Y	13	9	13
AGA64614.1	Y	Y	Y	Y	Y	Y	–	–	30	25	27
AGA64636.1	–	Y	Y	–	Y	–	–	–	29	34	25
AGA64650.1	–	Y	–	–	Y	Y	Y	Y	47	35	47
AGA64651.1	–	–	Y	Y	Y	Y	–	–	4	5	1
AGA64766.1	–	Y	Y	–	–	–	–	–			
AGA64823.1	–	Y	Y	Y	–	–	Y	Y	16	11	14
AGA64892.1	Y	Y	Y	Y	Y	Y	–	–			
AGA64980.1	–	Y	Y	Y	Y	Y	–	Y	61	68	68
AGA65052.1	Y	Y	Y	Y	Y	Y	–	–	54	47	61
AGA65105.1	–	Y	Y	–	Y	Y	Y	Y	6	4	6
AGA65110.1	–	Y	Y	Y	–	–	–	–			
AGA65128.1	–	–	–	–	–	–	Y	Y	5	2	1
AGA65134.1	–	–	–	–	–	–	–	Y	8	8	12
AGA65143.1	Y	Y	Y	Y	Y	Y	–	–	31	30	33
AGA65153.1	Y	Y	Y	Y	–	Y	–	–	5	3	0
AGA65154.1	–	Y	Y	Y	Y	–	–	–	10	9	9
AGA65155.1	–	–	–	–	–	–	Y	Y			
AGA65174.1	–	–	–	–	–	–	Y	Y			
AGA65230.1	–	–	–	–	–	–	Y	Y			
AGA65239.1	–	–	Y	Y	–	Y	–	–			
AGA65242.1	–	–	–	Y	–	–	–	Y	2	2	0
AGA65276.1	–	Y	Y	Y	Y	–	–	–	50	40	48
AGA65326.1	–	Y	–	Y	–	Y	–	–	2	1	3
AGA65331.1	–	Y	Y	Y	–	–	–	–	9	2	3
AGA65241.1	–	–	–	Y	–	Y	–	–	25	21	18

Note: Y represent the protein is predicted to have β barrel domain, localized in outer membrane compartment or is a lipoprotein based on sequence analysis from different predictors.

### Scanning electron microscopy

**Figure 1:**
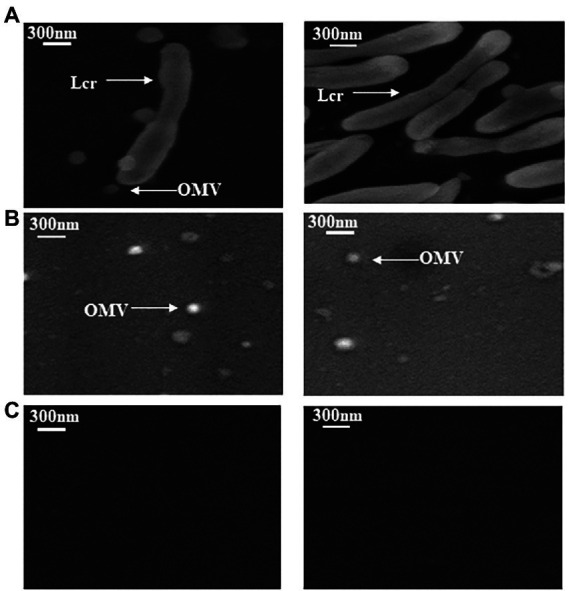
Scanning electron microscopy image of *Liberibacter crescens* BT-1 outer membrane vesicles. **(A)** Representative samples of Lcr cells. **(B)** Representative outer membrane vesicles (OMVs) extracted from Lcr. **(C)** Serum free BM7 medium. Each experiment contains three biological replicates and the experiment was repeated twice with similar results.

Outer membrane vesicles were isolated from Lcr BT-1. Lcr cells, OMVs and medium-only samples were observed under SEM. Bacterial cells and vesicle-like structures were found in bacteria samples. Vesicles were found in OMV samples but not in the medium (Figure 1), suggesting the producing of OMVs by Lcr. The diameter of 66 OMVs was measured and averaged 110 ± 7 nm (91 nm minimum and 120 nm maximum), which is consistent with previous report that OMVs are from approximately 20–350 nm in size (Turner et al., 2018).

### Identification of *Liberibacter crescens* outer membrane vesicle proteins *via* LC–MS/MS

Here, proteins were considered as OMV proteins if they were present in all three biological replicates. Consequently, a total of seven proteins were identified from OMV samples of Lcr (Table 3) including porin AGA65052.1, 25 kDa outer-membrane immunogenic protein precursor AGA65143.1, D-alanyl-D-alanine carboxypeptidase WP_051012132.1, homoserine dehydrogenase AGA64434.1, thioredoxin C-1 AGA65357.1, and two proteins with unknown function AGA64557.1 and AGA64826.1. These seven proteins were predicated to be in the outer membrane (2), inner membrane (1), extracellular (1) and cytoplasmic (2) compartments. Among the 7 OMV proteins of Lcr identified by LC–MS/MS, 5 have homologs in Las strains (Table 3).

**Table 3:** Proteins identified from *Liberibacter crescens* BT-1 outer membrane vesicle samples.

Accession number	Annotation	Subcellular localization prediction	Homolog in Las*
AGA65052.1	Porin	Outer membrane	WP_012778533.1
AGA65143.1	25 kDa outer-membrane immunogenic protein precursor	Outer membrane	WP_015452561.1
AGA64557.1	Protein with unknown function	Inner membrane	absent
WP_051012132.1	D-alanyl-D-alanine carboxypeptidase	Extracellular	WP_015452463.1
AGA64434.1	Homoserine dehydrogenase	Cytoplasmic	WP_015824937.1
AGA65357.1	Thioredoxin C-1	Cytoplasmic	WP_012778720.1
AGA64826.1	Protein with unknown function	unknown	absent

* Homolog accession # in Las psy62 was shown.

Subcellular localization prediction was conducted using predictor PSORTb, CELLO and SOSUI GtamN.

### Identification of *Liberibacter crescens* extracellular proteins *via* LC–MS/MS

Next, we investigated Lcr extracellular proteins. A protein was considered positive if it was present in all three biological replicates. A total of 26 proteins were identified from extracellular protein samples (Table 4) including 1 predicted extracellular protein, 5 outer membrane proteins, 7 periplasmic proteins, and 10 cytoplasmic proteins. Among the 26 putative extracellular proteins, 21 proteins were present in Las strains (Table 4).

**Table 4:** Proteins identified from *Liberibacter crescens* BT-1 extracellular protein samples.

Accession number	Annotation	Subcellular localization prediction	Homolog in Las
AGA65195.1	Chaperone protein DnaK	Cytoplasmic	WP_015824904.1
AGA65256.1	Phosphate ABC transporter, periplasmic phosphate-binding protein PstS	Periplasmic	WP_015452454.1
WP_041770579.1	Protein with unknown function	Cytoplasmic	ACT56857.1
AGA64093.1	Mitochondrial processing peptidase-like protein	Cytoplasmic	WP_012778683.1
AGA64249.1	Heat shock protein 60 family chaperone GroEL	Cytoplasmic	WP_015452683.1
AGA65048.1	Methionine ABC transporter substrate-binding protein	Cytoplasmic	absent
AGA64732.1	Dihydrolipoamide dehydrogenase of 2-oxoglutarate dehydrogenase	Cytoplasmic	WP_015452884.1
AGA65091.1	Enolase	Cytoplasmic	WP_171816669.1
AGA65268.1	Nonheme iron-containing ferritin	Cytoplasmic	WP_015452441.1
AGA64729.1	Glutathione reductase	Cytoplasmic	WP_015452886.1
AGA65203.1	Protein with unknown function	Cytoplasmic	absent
AGA64875.1	Glutamate Aspartate periplasmic binding protein precursor GltI	Periplasmic	WP_012778390.1
AGA64945.1	Flagellin protein FlaA	Extracellular	WP_015452627.1
AGA64040.1	Outer membrane protein assembly factor YaeT precursor	Outer membrane	WP_015452389.1
AGA65052.1	Porin	Outer membrane	WP_012778533.1
AGA65143.1	25 kDa outer-membrane immunogenic protein precursor	Outer membrane	WP_015452561.1
AGA64614.1	Protein with unknown function	Outer membrane	WP_015452561.1
AGA64650.1	Outer membrane lipoprotein Omp16 precursor	Outer membrane	WP_015452784.1
AGA64091.1	Branched-chain amino acid ABC transporter, amino acid-binding protein	Periplasmic	absent
AGA65006.1	Manganese ABC transporter, periplasmic-binding protein SitA	Periplasmic	WP_015452621.1
AGA64788.1	Zinc ABC transporter, periplasmic-binding protein ZnuA	Periplasmic	WP_015452621.1
AGA64318.1	Peptide/opine/nickel uptake family ABC transporter, periplasmic substrate-binding protein	Periplasmic	absent
AGA64342.1	TolB protein precursor	Unknown	WP_015452610.1
AGA64088.1	Branched-chain amino acid ABC transporter, amino acid-binding protein	Unknown	absent
AGA65071.1	L-proline glycine betaine binding ABC transporter protein ProX	Unknown	WP_012778561.1
AGA64598.1	Glutamate Aspartate periplasmic binding protein precursor GltI	Periplasmic	WP_012778390.1

Note: Homolog accession # in Las psy62 was shown.

Subcellular localization prediction was conducted using predictor PSORTb, CELLO, and SOSUI GtamN.

### Bioinformatic analysis of outer membrane proteins

Outer membrane proteins of 13 Liberibacter strains were analyzed using subcellular predictors, transmembrane beta barrel domain predictors and lipoprotein predictors (Supplementary Tables 2–14). The number of OMPs ranged from 33 to 65 for different strains and most *Ca.* Liberibacter strains have approximately 40 to 50 OMPs, representing about 3–7% of their total coding sequences (Supplementary Table 15). Among them, most strains have 20 to 30 hypothetical proteins as putative OMPs. The conserved outer membrane proteins like OmpA, and porins were also identified. Most strains have only 1 or 2 proteins identified with similar definitions. But there are a few exceptions: In *Lcr* BT-0, 4 outer membrane protein assembly factors were identified and 5 porins were identified.

Among the predicted OMPs, surface antigen protein, pilus assembly protein, OmpA family protein, and outer membrane lipoproteins exist in all five species. There are also some proteins that are species specific: Iron-dependent peroxidase, M23/M37 family peptidase, dihydrolipoamide dehydrogenase, thioredoxin reductase, 3-ketoacyl-ACP reductase, lysophospholipase, and opacity protein are only found in *Ca. L. americanus*. N-acetylglutamate synthase protein, alanine racemase protein, 3-oxoacyl-(acyl carrier protein) synthase II, monooxygenase FAD-binding protein, and HemY domain-containing protein are specific to *Ca. L. africanus*. *Liberibacter crescens* has many species-specific OMPs including: putative polysaccharide deacetylase, kinesin-like protein, 25 kDa outer-membrane immunogenic protein, glycosyl hydrolase, dual specificity protein, DUF5309 domain-containing protein, tail fiber domain-containing protein, glycoside hydrolase family 25 protein, SIMPL domain-containing protein, alpha/beta hydrolase, DUF3126 family protein, peptidoglycan DD-metalloendopeptidase family protein, AsmA family protein, and EAL domain-containing protein. For *Ca. L. americanus*, phage-related integrase/recombinase, putative peptidoglycan binding protein, peptidyl-prolyl cis-trans isomerase protein, putative membrane-bound lytic murein transglycosylase signal peptide protein, hydroxymethylglutaryl-CoA synthase, and phosphatidylcholine synthase are species specific. Compared to other four species, Las has very few different OMPs which include DUF2155 domain-containing protein and GlcNAc transferase. Las also has different outer membrane proteomes for different strains. Strain TaiYZ2, JXGC, JRPAMB1, CoFLP, and A4 are similar.

## Discussion

In this study, we investigated the proteins in the OM fraction of Liberibacter using both LC–MS/MS and bioinformatic approaches. Figty five proteins were identified in Lcr outer membrane fraction by LC–MS/MS. Protein BLAST results showed 52 of them have homologs in Las strains. Among these 55 proteins, 14 were also predicted to be OMPs by bioinformatic analyses and 13 proteins have been experimentally confirmed to localize to the outer membrane including YaeT (AGA64040.1; Stenberg et al., 2005), lipoprotein CmeC (AGA64980.1; Su et al., 2014), porin (AGA65052.1; Stenberg et al., 2005), lipoprotein ComL (AGA64591.1; Volokhina et al., 2009), translation elongation factor Tu (AGA64400.1; Harvey et al., 2019), GroEL (AGA64249.1; Rauch et al., 2021), and type II/IV secretion system secretin RcpA/CpaC (AGA65276.1; Clock et al., 2008). In addition, 32 predicted cytoplasmic proteins were also detected in the outer membrane compartment. Many cytoplasmic proteins were reported to traffic onto the cell surface or in extracellular secretions (Vanden Bergh et al., 2013). For instance, EF-Tu is primarily a cytoplasmic protein, but can localize to both the outer membrane and outer membrane vesicles of *Acinetobacter baumannii* (Harvey et al., 2019). GroEL is a known cytoplasmic protein, but was found to be an immunodominant surface-exposed antigen of *Rickettsia typhi* (Rauch et al., 2021). The surface-associated moonlighting proteins have been verified using diverse experimental approaches including florescence and electron microscopy (Bergmann et al., 2001; Candela et al., 2010; Yamaguchi et al., 2010; Robinson et al., 2013; Gründel et al., 2015; Jarocki et al., 2015). It is important to note that mass spectrometry plays instrumental roles in revealing the surface localization for proteins that are not predicted to reside on the cell (Jeffery, 2005; Robinson et al., 2013; Jarocki et al., 2015; Tacchi et al., 2016; Wang and Jeffery, 2016; Widjaja et al., 2017). However, we could not exclude the possibility of issues in extraction of outer membrane proteins. For example, some cytoplasmic proteins such as ribosomal proteins and RNA polymerases may result from contamination during the processing. Similar situation has also been found in *Ehrlichia ruminantium*, *Pseudomonas aeruginosa* and *Yersinia ruckeri* (Coquet et al., 2005; Seyer et al., 2005; Moumène et al., 2015).

According to the bioinformatic analyses, most Liberibacter strains have 40–50 putative OMPs. For Lcr, 50 OMPs were predicted including the 14proteins identified in all 3 replicates with an average ≥10 spectrum counts/replicate in the LC–MS/MS data. In addition, another12 predicated OMPs also had some spectrum counts in the LC–MS/MS data. In total, 95 different proteins were found in the 13 Liberibacter strains investigated in this study. Among them, 42 proteins have been experimentally verified in other gram-negative bacteria. In *E. coli,* BAM complex consists of five outer membrane assembly factors BamA, BamB, BamC, BamD, and BamE. The complex is embedded in the outer membrane and it folds and inserts integral β-barrel proteins in the outer membrane (Sandoval et al., 2011). BamC and BamE were also found in the outer membrane of *Aeromonas hydrophila* (Lin et al., 2018). Outer membrane protein YaeT is required for membrane protein assembly in *E. coli* (Werner and Misra, 2005). Organic solvent tolerance protein OstA was reported to be an outer membrane-associated protein in *E. coli* and it can contribute to n-hexane resistance of bacteria (Abe et al., 2003). OmpA is a conserved porin protein and it has been found in many bacteria such as *Helicobacter pylori*, *Escherichia coli*, *Yersinia ruckeri*, and *Aeromonas hydrophila* (Molloy et al., 2000; Carlsohn et al., 2006; Lin et al., 2018; Ormsby et al., 2019). OmpA can mediate bacterial biofilm formation, cell infection, immunomodulation and antibiotic resistance (Nie et al., 2020). Omp25 is a conserved outer membrane protein. Omp25 from *Brucella ovis* can be exported to the outer membrane of *E. coli* (Lintermans et al., 1996). Omp25 also affects the penetration and survival of *Brucella ovis* inside host cells (Caro-Hernández et al., 2007). TadD protein was reported to be involved in the assembly of pilus in *Aggregatibacter actinomycetemcomitans* and it can be found in both the outer membrane and inner membrane of bacteria (Clock et al., 2008). Gram-negative bacteria are capable of expelling substrates from within the cell using three-component efflux pumps, which span the inner and outer membrane and the periplasmic space (Lin et al., 2002; Xu et al., 2012).

Many lipoproteins were characterized to be outer membrane proteins in other bacteria. For example, peptidoglycan-associated lipoprotein (Pal) was identified by LC–MS/MS from outer membrane fraction of *Helicobacter pylori* (Carlsohn et al., 2006). Pal is a protein anchored to out membrane of bacteria and it can interact with Tol proteins to form Tol-Pal complex. Tol-Pal proteins were reported to affect the transportation of compounds through cytoplasm membrane, the amount of outer membrane vesicle produced and pathogenicity in bacteria (Godlewska et al., 2009). Outer membrane protein OmlA, Omp10, Omp16, Omp19 are lipoproteins (Ochsner et al., 1999; Tibor et al., 1999). OMP rare lipoprotein A from *Pseudomonas aeruginosa* contributes to the separation of daughter cells and maintenance of rod shape (Jorgenson et al., 2014). Outer membrane lipoprotein YfiO functions as a part of a multiprotein complex which is required for outer membrane protein assembly (Wu et al., 2005). Competence lipoprotein ComL from *Neisseria meningitidis* is an outer membrane protein with DNA binding properties (Benam et al., 2011). Chaperone proteins were found in the outer membrane of *Ehrlichia ruminantium* and *Aeromonas hydrophila* (Moumène et al., 2015; Lin et al., 2018). Chaperones aid in protein folding and transporting proteins in cytoplasm and across cell membrane. In *Borrelia burgdorferi,* chaperon protein Hsp60 was detected both in cytoplasm and cell envelop (Scorpio et al., 1994). An OMP from *Neisseria meningitidis* was reported to be a M23 family peptidase (Wang et al., 2011). Surface antigen D15 was identified from outer membrane of *Helicobacter pylori* (Carlsohn et al., 2006). Surface antigen D15 from *Haemophilus influenzae* has been shown to be a target of host immunity (Flack et al., 1995). A lysophospholipase VolA (*Vibrio* outer membrane lysophospholipase A) from *Vibrio cholerae* was reported to be a surface-exposed lipoprotein phospholipase (Pride et al., 2013). Opacity-associated proteins are OMPs which function in the adhesion of bacteria (De Jonge et al., 2002). Translocation protein TolB is a component of Tol-dependent translocation system in bacteria. It can form a complex with Pal and associate with the outer membrane (Abergel et al., 1999). TolB was identified by mass spectrometry from outer membrane samples of *Pasteurella multocida* (Prasannavadhana et al., 2014). Murein transglycosylase can degrade bacterial cell well murein (Hoeltje et al., 1975). Multiple murein transglycosylases were identified in *E. coli* outer membrane samples (Molloy et al., 2000). lipopolysaccharide (LPS) assembly protein LptD is an OMP which can translocate LPS from the periplasm across the outer membrane (Lundquist and Gumbart, 2020).

Some predicted OMPs might trigger plant immune responses, consistent with HLB being a pathogen-triggered immune disease. Similar proteins have been reported to be involved in pathogenicity and immunity. Bacterial lysophospholipases can function as virulence factors. Sphingomyelinase Ds have lysophospholipase D activity and can generate lysophosphatidic acid. Aggregation of lysophosphatidic acid in blood can induce platelet aggregation and endothelial barrier dysfunction (Flores-Díaz et al., 2016). Recognition of lipoprotein from human pathogen *Staphylococcus aureus* is required for host defense against bacteria (Wardenburg et al., 2006).

We have demonstrated that Lcr forms outer membrane vesicles, providing the first experimental evidence that Liberibacter species including Las may use OMVs to transport virulence factors during interactions with plant hosts. Seven OMV proteins were identified by LC–MS/MS. Among them, porins have been found in OMV of *E. coli* and porins are involved in regulating the permeability of β-lactam antibiotics. Transferring β-lactam antibiotics into outer membrane vesicles *via* porins and degrading them was suggested as a strategy for bacteria to avoid the effect of antibiotics (Kim et al., 2020). β-lactam antibiotics such as ampicillin and cefalexin were reported to suppress Las infection and HLB development (Zhang et al., 2014; Yang et al., 2020, 2022). Antibiotic application plus outer membrane vesicle control might improve efficacy for HLB management. D-alanyl-D-alanine carboxypeptidase was identified as an OMV protein in Lcr BT-1 and one zinc uptake-regulator (Zur)-regulated lipoprotein A (ZrlA) from *Acinetobacter baumannii* was reported to have enzymatic activity of D-alanyl-D-alanine carboxypeptidase. The ZrlA-deficient mutant strain produced 9.7 times more OMVs than the wide type strain and the OMVs generated by the mutant were more cytotoxic (Kim et al., 2021). Thioredoxin C-1 was identified in Lcr BT-1 as an OMV protein and one thioredoxin-related protein was also found in *Neisseria meningitidis* outer membrane vesicles (Lappann et al., 2013). Thioredoxin A (TrxA) from *Acinetobacter baumannii* is a virulence factor. OMVs isolated from TrxA-deficient bacteria resulted in increased lung permeability in mouse compared to wild-type bacteria (Shrihari et al., 2022). Five of these LC–MS/MS identified OMV proteins have homolog proteins in Las and it remains to be determined how they are involved in interactions with citrus plants.

A total of 26 proteins were identified from the extracellular compartment of Lcr BT-1. Porin protein AGA65052.1 and 25 kDa outer-membrane immunogenic protein precursor AGA65143.1 were also detected from outer membrane fraction and OMV samples. Porin was reported to be secreted by spheroplasts of *E. coli* cells (Sen and Nikaido, 1990). Heat shock protein, GroEL was found in membrane fraction of *Clostridium difficile* and in the extracellular space after heat stress. It serves an adhesive function in this bacteria (Hennequin et al., 2001). α-enolase from *Streptococcus pneumoniae* can induce the formation of neutrophil extracellular traps and cause cell death of human neutrophils (Mori et al., 2012). Among these 26 proteins, 21 have homolog proteins in Las. Because Liberibacter species do not have specific protein secretion systems such as type II and III, it remains to be determined how the identified proteins are present in the extracellular compartment. One possibility is that some proteins remain intact after death of bacterial cells.

## Summary

In summary, we have used Lcr as a surrogate to investigate the outer membrane proteins, OMV proteins and proteins in the extracellular compartments of *Ca. Liberibacter* species. The roles of these proteins in activating plant immune responses have not been reported previously. Because Las colonizes inside sieve element cells and HLB is a pathogen-triggered immune disease, it is possible that citrus cells can recognize proteins in the OM fraction, OMV proteins and extracellular proteins directly. This study advances our understanding of the biology of *Ca.* Liberibacter species and identifies many putative proteins that might play critical roles in interactions with host proteins in the phloem tissues.

## Data availability statement

The datasets presented in this study can be found in online repositories. The names of the repository/repositories and accession number(s) can be found at: MassIVE MSV000089995.

## Author contributions

NW designed the experiments. YH did the outer membrane proteome analysis and isolated proteins from *Liberibacter crescens*. YH and DS conducted scanning electron microscopy. FZ, JK performed LC–MS/MS and database searching. SC supervised the LC–MS/MS work. YH and NW wrote the manuscript. All authors contributed to the article and approved the submitted version.

## Funding

The research has been supported by Florida Citrus Initiative, Florida Citrus Research and Development Foundation, USDA National Institute of Food and Agriculture grant # 2018-70016-27412, #2016-70016-24833, and #2019-70016-29796.

## Conflict of interest

The authors declare that the research was conducted in the absence of any commercial or financial relationships that could be construed as a potential conflict of interest.

## Publisher’s note

All claims expressed in this article are solely those of the authors and do not necessarily represent those of their affiliated organizations, or those of the publisher, the editors and the reviewers. Any product that may be evaluated in this article, or claim that may be made by its manufacturer, is not guaranteed or endorsed by the publisher.
